# Strongyloidiasis mimics duodenal lymphoma in a patient with systemic lupus erythematosus and antiphospholipid syndrome: a case report

**DOI:** 10.1186/s13256-024-04914-4

**Published:** 2024-12-25

**Authors:** Ayoub Basham, Sanaz Soleimani, Atash Ab Parvar, Arash Rahimi, Ebrahim Evazi, Seyed Hamid Moosavy

**Affiliations:** 1 Student Research Committee, Faculty of Medicine, Hormozgan University of Medical Sciences, Bandar Abbas, Iran; 2 Department of Internal Medicine, Shahid Mohammadi Hospital, Hormozgan University of Medical Sciences, Bandar Abbas, Iran; 3 Department of Pathology, Shahid Mohammadi Hospital, Hormozgan University of Medical Sciences, Bandar Abbas, Iran; 4 Department of Gastroenterology, Shahid Mohammadi Hospital, Hormozgan University of Medical Sciences, Bandar Abbas, Iran; 5 Department of Pulmonology, Shahid Beheshti University of Medical Science, Tehran, Iran

**Keywords:** *Strongyloides stercoralis*, Systemic lupus erythematous, Antiphospholipid syndrome, Duodenal lymphoma, Non-Hodgkin lymphoma, Case report

## Abstract

**Background:**

Systemic lupus erythematosus is a multi-organ autoimmune disorder that is treated by immunosuppressive agents that weaken the immune defense against opportunistic pathogens and latent infections such as strongyloidiasis. Herein, we report the case of a 43-year-old woman known to have systemic lupus erythematosus who presented with gastrointestinal symptoms, edema, and bone pain 2 months after receiving immunosuppressive treatment.

**Case presentation:**

A 43-year-old Iranian female known to have systemic lupus erythematosus and antiphospholipid syndrome presented with abdominal pain, nausea, vomiting, and generalized edema. She was on CellCept, prednisolone, and hydroxychloroquine. The vital signs were within the normal range. On physical examination, no rash was observed on the skin. There was only a mild tenderness in epigastric region. The results of blood analysis revealed hypochromic microcytic anemia, normal leukocyte count with mild eosinophilia. Liver enzymes as well as renal function tests were within the normal range. Stool examination was negative for trophozoites, ova, or cysts of parasites. Endoscopic findings included a generalized nodular appearance of duodenum with an infiltrative mucosa in the wall of duodenum, suggesting lymphoma. The pathology report determined the diagnosis of strongyloidiasis. Cap albendazole 400 mg was administered twice a day for 2 weeks. Abdominal pain was completely relieved 1 week after starting the treatment. The patient was eventually discharged after 10 days.

**Conclusion:**

The significance of this case report is the necessity to send complete blood count and serologic assays to screen latent strongyloidiasis before receiving immunosuppressive agents in patients with systemic lupus erythematosus.

## Introduction

Systemic lupus erythematous is a multi-organ autoimmune disorder commonly affecting young females that causes serious sequelae if left untreated [1]. Corticosteroids and other immunosuppressive agents act like a double-edged sword. Although immunosuppressive agents are pillars of SLE treatment, they make the patients susceptible to opportunistic pathogens, reactivation of latent infections, and developing more serious conditions such as cancer by interrupting the cellular and humoral immune function [2–4]. Strongyloidiasis is a condition caused by *Strongyloides stercoralis* (SS), a soil-transmitted nematode with both a living-free and living-dependent life cycle, which is a public health concern in tropical regions, rural, and low-socioeconomic areas [5]. Strongyloidiasis is commonly asymptomatic; however, in immunocompromised conditions, such as receivers of organ transplantation, autoimmune patients on immunosuppressive treatments, and infection by human immunodeficiency virus (HIV), it may present with gastrointestinal (GI) and respiratory symptoms, including abdominal cramps, nausea, vomiting, malabsorption, weight loss, GI bleeding, cough, asthma-like symptoms, and dyspnea [6, 7]. We report herein the case of a 43-year-old woman known to have SLE and antiphospholipid syndrome who presented with GI symptoms and edema with endoscopic evidence suggesting duodenal lymphoma that turned out to be SS infection on pathology assessment.

## Case presentation

A 43-year-old Iranian female known to have SLE and antiphospholipid syndrome for about 2 months referred to a tertiary hospital complaining of abdominal pain, nausea, vomiting, and generalized edema 1 month after receiving immunosuppressive treatment for her autoimmune condition. Abdominal pain was described as a burning sensation in epigastric region with constant, nonradiating, nonpositional features that gradually intensified 30 min after having solid and liquid meals. Edema had progressively worsened in the last month. The edema was described as a nondependent edema affecting periorbita and extremities. She was hospitalized twice in a 1-month interval with the same presentation. In her first admission, the endoscopic evidence was in favor of candidiasis esophagitis and *Helicobacter pylori*-induced gastritis. Her symptoms improved after administrating drop nystatin, oral fluconazole, and four-drug regimens for *H. pylori* eradication. Two weeks later, she was admitted for the second time and received parenteral pantoprazole based on the previous diagnosis of gastritis; however, the symptoms relapsed a few days later after relative improvement.

The past medical records indicate involvement with SLE and antiphospholipid syndrome, as confirmed by serologic tests showing high titers of antinuclear antibody (ANA), elevated levels of anti-double-strand DNA (anti-ds DNA), and anti-cardiolipin antibodies (IgG). The protein-to-creatinine ratio of 1.7 was in favor of a lupus nephropathy, as well (Table 1). She had been receiving CellCept 1 mg every 12 h, prednisolone 5 mg daily, hydroxychloroquine 200 mg daily, and acetylsalicylic acid (ASA) 80 mg daily for the last 2 months since the diagnosis of SLE. Her social history was free of smoking, alcohol, and drugs. The familial history was negative for malignancy. She did not mention a recent travel history to endemic areas inside the country or abroad.

**Table 1:** Laboratory workup for systemic lupus erythematous and antiphospholipid syndrome

Parameter	Value	Reference
Serologic tests		
ANA (Titer)	1:320	> 1:80 positive
Anti-ds DNA (IU/mL)	> 240.0	> 25.0 positive
LA (seconds)	29.9	33–40
Anti-cardiolipin Ab (IgG) (GPL U/mL)	60.6	> 18.0 positive
Anti-cardiolipin Ab (IgM) (MPL U/mL)	< 2.0	> 15.0 positive
Anti-B_2_-glycoprotein Ab (IgG) (U/ mL)	0.90	> 20.0 positive
Anti-B_2_-glycoprotein Ab (IgM) (U/mL)	< 3.0	> 18.0 positive
U/A		
Protein (mg/dL)	211.0	0–15
Creatinine (mg/dL)	124.15	28–217
Protein/creatinine ratio	1.69	≥ 0.3 positive

^ANA, antinuclear antibody; Anti−ds DNA, anti−double strand DNA; LA, lupus anticoagulant; U/A, urine analysis^

She was ill but not toxic in general appearance. The vital signs were as follows: pulse rate, 87 per minute; blood pressure, 100/65 mmHg; respiratory rate, 14 per minute, and body temperature, 36.6 °C. On physical examination, no rash was observed on the skin. Conjunctiva was mild pale, and sclera was not icteric. Periorbita had mild edema. No ulcer, thrush, or other lesions were seen in tongue, buccal, and pharynx mucosa. No lymphadenopathy existed in cervical, axillary, supraclavicular, or inguinal region. No rale or wheezing was auscultated in the lungs. On abdominal examination, abdomen was not distended and bowel sounds were normal. There was mild tenderness in epigastric region. All quadrants were tympanic on percussion. Shifting dullness was negative for ascites. In extremities, there was nondependent, pitting edema. Capillary refill was prolonged, and limbs were cold.

Blood, urine, and stool samples were sent to a laboratory for further evaluation. The results of blood analysis revealed hypochromic microcytic anemia with increased reticulocyte distribution width (RDW), high ferritin level, normal leukocyte count with mild eosinophilia, mild thrombocytosis, normal renal function test, normal liver enzymes, and liver function tests, decreased protein and albumin levels, normal thyroid function test, and negative results for celiac antibodies titer (Table 2). The result of urine analysis (U/A) was normal. Stool examination was negative for undigested food, fat drops, occult blood, trophozoites, ova, or cysts of parasites. The result of stool culture was negative for salmonella and other pathogenic bacteria.

**Table 2:** Laboratory data in first and current admission

Parameter	First admission	Current admission	Reference range
WBC (10^3^cells/µl)	14.5	8.5	4–11
Neutrophils (%)	79.3	52.5	
Lymphocytes (%)	13.8	28.9	
Monocytes (%)	2	11.4	
Eosinophils (%)	4.5	6.8	
Basophils (%)	0.4	0.4	
Hb (mg/dL)	10.5	9.4	12–16
MCV (fl)	74.6	76.6	80–100
MCH (pg)	24.9	25.3	27–34
RDW (%)	16.8	18.7	11–16
Ferritin (ng/ml)	—	218.3	5–204
Platelets (10^3^cells/µl)	817	617	150–450
ESR	85	–	< 15
CRP	2+	—	Negative
BUN (mg/dL)	15.4	7	6–20
Creatinine (mg/dL)	0.6	0.43	0.5–1.25
Sodium	132	–	135–145
Potassium	4.8	–	3.5–5.2
BS	52	–	70–100
CPK	13	–	10–120
LDH	286	–	140–280
Total bilirubin (mg/dL)	0.5	0.21	0.1–1.2
Direct bilirubin (mg/dL)	0.1	0.07	< 0.3
AST (IU/L)	30	25.63	5–32
ALT (IU/L)	25	25.69	5–33
ALP (IU/L)	165	151	65–305
Amylase (IU/L)	75.83	–	28–100
Lipase (IU/L)	16.5	–	13–60
Protein (g/dL)	–	4.79	6.8–7.3
Albumin (g/dL)	–	2.40	3.5–5.2
IgA total (g/L)	–	2.31	0.7–4
Anti-TTG (IU/mL)	–	1.0	Positive ≥ 20
T4 (µg/dL)	–	6.94	4.6–14.1
TSH (µIU/mL)	–	1.94	0.27–5.39
U/A			
Color	Yellow	Yellow	
Appearance	Clear	Semiclear	
Specific gravity	1.030	1.025	
pH	5	5	
Protein	Trace	Negative	
Glucose	Negative	Negative	
Ketone	Positive	Negative	
Bilirubin	Negative	Negative	
Urobilinogen	Negative	Negative	
Blood/Hgb	Negative	Negative	
Nitrite	Negative	Negative	
WBC	1–2	4–5	
RBC	1–2	0–1	
EP	2–3	4–5	
Bacteria	Negative	Rare	
Mucus	Negative	Moderate	
Casts	Negative	Negative	
Crystals	Few Ca-oxalate	Negative	
Yeast	Negative	Negative	
S/E			
Consistency	Soft	Formed	
Color	Brown	Brown	
Fat	Not seen	Not seen	
Undigested food	Not seen	Not seen	
RBC/hpf	Not seen	Not seen	
WBC/hpf	Not seen	Not seen	
Ova	Not seen	Not seen	
Cyst	Not seen	Not seen	

WBC, white blood cells; Hb, hemoglobin; MCV, mean corpuscular volume; MCH, mean concentration of hemoglobin; RDW, red cell distribution width; ESR, erythrocyte sedimentation rate; CRP, C-reactive protein; BUN, blood urea nitrogen; BS, blood sugar; CPK, creatine phosphokinase; LDH, lactate dehydrogenase; AST, aspartate transaminase; ALT, alanine transaminase; ALP, alkaline phosphatase; Anti TTG, anti-tissue transglutaminase; T4, thyroxine; TSH, thyroid stimulating hormone; U/A; urine analysis; S/E, stool exam

**Fig. 1:**
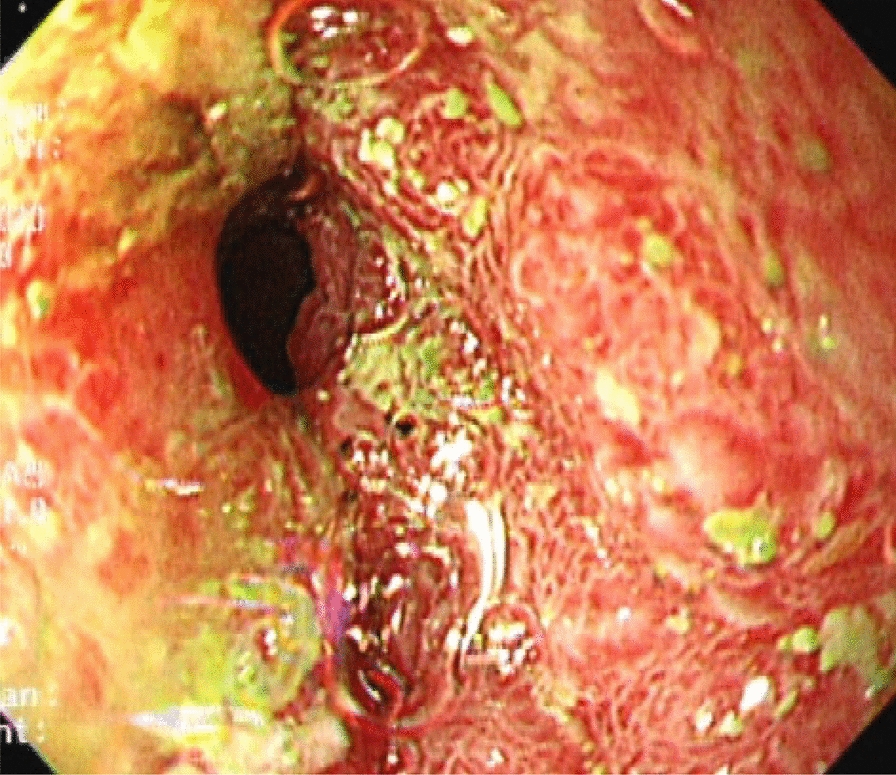
Endoscopic view of the duodenum showing a generalized nodular appearance with infiltrative mucosa in the anterior wall of the bulb and second part of the duodenum and distal portion of D2 stenosis

**Fig. 2:**
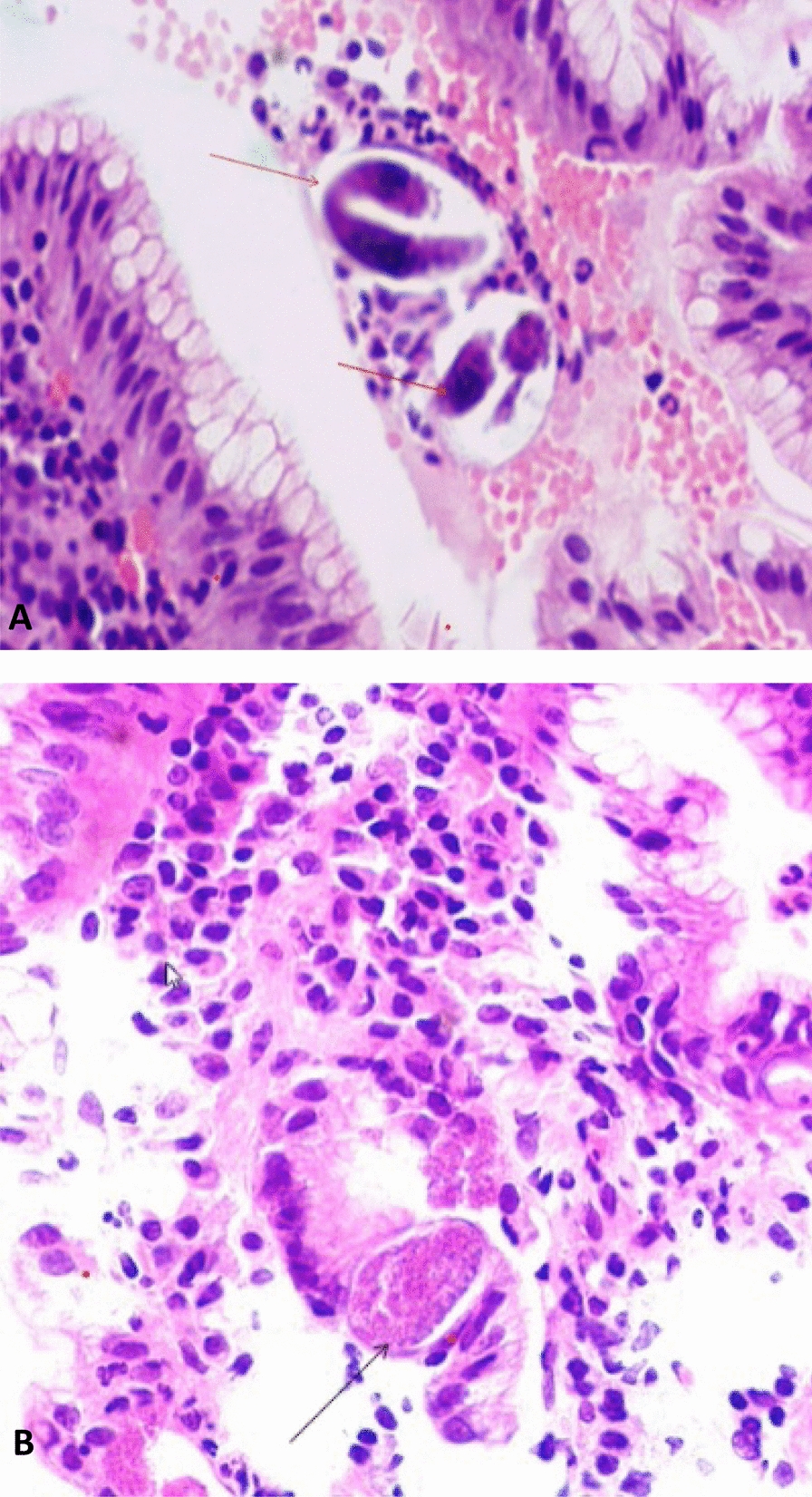
Hematoxylin and eosin staining of duodenal tissue reveals the moderate infiltration of inflammatory cells, including lymphocytes, neutrophils, and plasma cells in this high-power view. **A** The arrows point to a curved larva with a pointed tail, which is diagnostic for *Strongyloides stercoralis*. **B** The points to a *Strongyloides* egg

Endoscopic findings revealed a generalized nodular appearance of duodenum with an infiltrative mucosa in the anterior wall of the bulb, second part of the duodenum, and distal portion of D2 stenosis, suggesting lymphoma (Fig. 1). The pathology report stated that villi and crypts had normal architectures, and lamina propria was edematous and moderately infiltrated by acute and chronic inflammatory cells with permeation of neutrophils in crypts and villi. Additionally, SS eggs and larvae were also seen within the crypts and surface epithelium. There were no evidence of malignancy and lymphoma in this specimen (Fig. 2).

Although ivermectin is the choice for treating strongyloidiasis, we treated with cap albendazole 400 mg twice a day for 2 weeks, owing to shortage of ivermectin in our hospital. Moreover, four vials of albumin were also given to the patient every 12 hours for 2 days. Abdominal pain relieved partially in the third day and completely in the seventh day of antihelminth therapy. Edema was significantly decreased in third day and completely relieved in the seventh day of albumin administration. After three consecutive negative results for stool examination, the patient was discharged on the tenth day of receiving treatment. The patient was visited 4 weeks after discharge, when she was in good condition.

## Discussion and conclusions

Strongyloidiasis is the fourth most common nematode infection, affecting approximately 613 million individuals worldwide, of whom 39.4 million live in the East Mediterranean region [8]. Strongyloidiasis occurs sporadically in the majority of Iran’s territory; however, Mazandaran Province in the north and Khuzestan Province in the south are endemic areas for SS infection [9, 10]. Our patient resided in Hormozgan Province in the south of Iran, which is not an endemic area for SS infection and does not have a common border with Khuzestan Province. Moreover, she did not mention a recent travel history to endemic areas in her home country or abroad.

The life cycle of SS in the host body commonly begins when filariform larva penetrates the skin while the host is walking bare foot, causing an itchy rash called larva currens [11]. Filariform larvae migrate to the lungs via the circulation, where they pass a part of their maturation and may present with coughing, asthma, dyspnea, and pneumonia. After maturation in the lungs, the larvae will be coughed up and swallowed to continue their maturation process in intestinal crypts and villi. In this stage, the infection may manifest with GI symptoms such as abdominal cramping, nausea, vomiting, abdominal bloating, watery diarrhea, and sometimes constipation. Female adults eventually lay eggs in intestinal crypts and villi, which hatch into rhabditiform larvae [6, 11]. Rhabditiform larvae are not infectious until they turn into filariform larvae again, which pass through feces and continue their free-living life or penetrate colon mucosa or perianal skin and begin the life cycle in the host body, a phenomenon called autoinfection, which may raise the load of infection in the intestine, causing complete or partial bowel obstruction [12].

Strongyloidiasis presents in three phases, viz. acute, chronic, and hyperinfection or disseminated. In the acute phase, the symptoms vary depending on the stage of the SS life cycle, such as skin rash, asthma-like symptoms, dyspnea, and GI symptoms. In the chronic phase, patients are commonly asymptomatic or mildly symptomatic. The GI system is commonly involved, presenting with abdominal discomfort, borborygmus, intermittent vomiting, constipation, or diarrhea. Eosinophilia is usually the only finding in asymptomatic patients [13, 14]. Hyperinfection syndrome, which occurs in 1.5–2.5% of strongyloidiasis cases, refers to countless number of multiplications and migration of larvae in an immunosuppressed state, which increases the number of larvae in the stool and/or sputum, leading to more severe intestinal and respiratory symptoms [6, 15].

One month after initiating immunomodulatory agents, abdominal pain, nausea, and vomiting began. She never complained of respiratory symptoms or skin rash in any of the three admissions. Looking at Table 2, note that neutrophilia occurred in the first hospitalization. Indeed, she was in the acute phase, causing an acute immune reaction with leukocytosis with neutrophil dominance [16]. Following misdiagnosis and mistreatment, the patient entered the chronic phase, where eosinophilia became notable. In the course of the disease, since initiation to last admission, the patient was not febrile and septicemic, thus chronic strongyloidiasis is a more accurate diagnosis than hyperinfection. Hypoalbuminemia is a common feature in chronic strongyloidiasis [17]. The patient’s edema gradually worsened owing to consumption of nutrients by the high load of SS worms.

Stool examination is the conventional method for diagnosis of strongyloidiasis. Single stool examination fails to detect larvae in up to 70% of cases. Several factors are involved in the low sensitivity of the stool examination method, such as a low parasitic load and irregular larval output. Several immunodiagnostic assays have been introduced, but they are ineffective in disseminated infections and have significant cross-reaction with hookworms, schistosomes, and filariae [18, 19]. SS is a rare parasite in our province, and we lacked laboratory kits for detecting SS antigens. Consistent with previous findings, sending stool for examination for one time has a high false negative rate and makes diagnosis a bit more challenging.

Endoscopic evaluation revealed irregular nodular lesions scattered all over the duodenal mucosa, suggesting duodenal-type follicular (D-FL) lymphoma. Non-Hodgkin’s lymphoma occurs 5.40 times more than in general population, because of immune dysregulation seen in autoimmune disease and administration of immunosuppressive agents [4, 20]. D-FL is recognized as a new entity in the World Health Organization classification update in 2016, accounting for 1–4% of gastrointestinal non-Hodgkin lymphomas. D-FL appears to have a close association with extranodal marginal zone lymphoma, which originates from mucosa-associated lymphoid tissue. Unlike LFs that are diagnosed at an advanced stage, D-FL is almost always diagnosed at a low stage and tends to be localized to the small bowel, specifically the second portion of the duodenum, thus the majority of oncologists watch and wait owing to its good prognosis [21, 22]. Follicular lymphomas are incidentally detected on endoscopy, appearing as single or multiple nodules or polypoid lesions with size of 1–5 mm. In this circumstance, biopsy is indicated from the lesions and surrounding tissue for better diagnostic yield [23]. Eventually, the results of biopsy determined the diagnosis in our challenging case.

The treatment of choice for SS infection in immunocompromised patients, specifically those who face hyperinfection syndrome, is ivermectin at dosage of 200 µg/kg/day administered orally for 2 days, and repeated every 2 weeks for three times, which yields a cure rate above 90% [24]. Results of a randomized trial showed that a single dose of 200 µg/kg of ivermectin or 400 mg/day for 3 days of albendazole for treatment of strongyloidiasis resulted in cure rates of 83% and 45%, respectively [25]. In cases that are refractory to treatment, therapy should be continued until stool smears are cleared of eggs and larvae, which takes at least 2 weeks [24]. The treatment of our patient with cap albendazole 400 mg twice a day for 2 weeks was successful.

In summary, we report herein a patient with SLE who complained of GI symptoms for 1 month, which started after a 1-month period following administration of corticosteroid and immunosuppressive agents. The clinical and laboratory evidence was not conclusive. Endoscopy view of duodenum was suggestive of duodenal lymphoma, however biopsy reports determined the diagnosis of strongyloidiasis. The significance of reporting this case pertains to the necessity of sending complete blood count, serologic assays, and multiple stool examinations to screen latent strongyloidiasis in candidates for receiving immunosuppressive agents for organ transplantation, blood malignancies, SLE, and other autoimmune diseases.

## Data Availability

Not applicable.

## Abbreviations

SLE: Systemic lupus erythematosus
CRP: C-reactive protein
SS: *Strongyloides stercoralis*
D-FL: Duodenal-type follicular
